# Statin Use and Markers of Immunity in the Doetinchem Cohort Study

**DOI:** 10.1371/journal.pone.0077587

**Published:** 2013-10-16

**Authors:** Hilda J.I. De Jong, Jan G.M.C. Damoiseaux, Rob J. Vandebriel, Patrick C. Souverein, Eric R. Gremmer, Mia Wolfs, Olaf H. Klungel, Henk Van Loveren, Jan Willem Cohen Tervaert, W.M. Monique Verschuren

**Affiliations:** 1 Laboratory for Health Protection Research, National Institute for Public Health and the Environment, Bilthoven, The Netherlands; 2 Department of Toxicogenomics, Maastricht University Medical Centre, Maastricht, The Netherlands; 3 Division of Pharmacoepidemiology and Clinical Pharmacology, Department of Pharmaceutical Sciences, Faculty of Sciences, Utrecht Institute for Pharmaceutical Sciences, *Utrecht* University, Utrecht, The Netherlands; 4 Central Diagnostic Laboratory, Maastricht University Medical Centre, Maastricht, The Netherlands; 5 Immunology, Maastricht University Medical Centre, Maastricht, The Netherlands; 6 Centre for Nutrition, Prevention and Health Services, National Institute for Public Health and the Environment, Bilthoven, The Netherlands; University of Ottawa, Canada

## Abstract

It has been suggested that statins can both stimulate and suppress the immune system, and thereby, may influence autoimmune diseases. Therefore, we studied effects of statins on innate and adaptive immunity, and self-tolerance by measuring serological levels of C-reactive protein (CRP), neopterin, immunoglobulin E (IgE) antibodies and the presence of autoantibodies (antinuclear antibodies (ANA) and IgM rheumatoid factor (RF)) in the general population. We conducted a nested case-control study within the population-based Doetinchem cohort. Data from health questionnaires, serological measurements and information on medication from linkage to pharmacy-dispensing records were available. We selected 332 statin users (cases) and 331 non-users (controls), matched by age, sex, date of serum collection, history of cardiovascular diseases, diabetes mellitus type II and stroke. Multivariate regression analyses were performed to estimate effect of statins on the immune system. The median level of CRP in statin users (1.28 mg/L, interquartile range (IQR): 0.59-2.79) was lower than in non-users (1.62 mg/L, IQR: 0.79-3.35), which after adjustment was estimated to be a 28% lower level. We observed an inverse association between duration of statin use and CRP levels. Elevated levels of IgE (>100 IU/mL) were more prevalent in statin users compared to non-users. A trend towards increased levels of IgE antibodies in statin users was observed, whereas no associations were found between statin use and levels of neopterin or the presence of autoantibodies. In this general population sub-sample, we observed an anti-inflammatory effect of statin use and a trend towards an increase of IgE levels, an surrogate marker for Th (helper) 2 responses without a decrease in neopterin levels, a surrogate marker for Th1 response and/or self-tolerance. We postulate that the observed decreased inflammatory response during statin therapy may be important but is insufficient to induce loss of self-tolerance.

## Introduction

Numerous clinical trials have demonstrated that statins, or anti-3-hydroxy-3-methylglutaryl-coenzyme A reductase (HMGCR) inhibitors, are effective in the primary and secondary prevention of cardiovascular diseases [1-3]. In addition to lowering cholesterol, statins have anti-inflammatory and immunomodulatory properties which also may contribute to the beneficial effects of these drugs [4,5]. Several studies of patients with rheumatoid arthritis (RA) and experimental studies in collagen-induced arthritis suggested beneficial effects of statins in arthritis [6-12]. Similarly, in studies of antiphospholipid syndrome, vasculitis, systemic sclerosis, and systemic lupus erythematosus (SLE) patients and murine lupus models, attenuation of lupus activity has been ascribed to statins [13-20]. On the other hand, it has been postulated that statins may facilitate the loss of self-tolerance, potentially resulting in autoimmune diseases. Indeed, several case-reports and reviews have linked statin use with autoimmune disorders, such as lupus-like syndrome, vasculitis, poly- and dermatomyositis, and necrotising autoimmune myositis [21-25]. Furthermore, we recently demonstrated that statins are associated with an increased risk of developing RA, SLE and polymyalgia rheumatica [26-29].

The proposed mechanisms by which statins may facilitate and/or be protective for the development of autoimmune diseases are unclear. As described in various studies, statins seem to affect the functions of immune cells, including natural killer cells, monocytes, macrophages and T cells [30]. Statins have been shown reduce pro-inflammatory cytokines, including tumor necrosis factor (TNF)-α, interleukin (IL)-6 and IL-8, and the levels of C-reactive protein (CRP), a marker of the innate immune system reflecting underlying systemic inflammation [31-35]. Statins may also block stimulation of T cells and inhibit interferon (IFN)-gamma induced macrophage activation [30], resulting in suppressed secretion of neopterin [36]. In a murine model mimicking multiple sclerosis, atorvastatin promoted the differentiation of Th (helper) 0 into Th2 cells, resulting in the systemic production of Th2 cytokines (IL-4, IL-5, IL-10, and transforming growth factor beta (TGF-β) [37]. IL-4 is a cytokine that induces differentiation of naive helper T cells (Th0 cells) to Th2 cells. Upon activation by IL-4, Th2 cells subsequently produce additional IL-4 which is responsible for the induction of immunoglobulin E (IgE) synthesis by B cells. Levels of circulating IgE antibodies are representative for Th2 immune responses [38,39], whereas neopterin is considered to be a marker of Th1 responses [36]. It has been suggested that statins may induce a shift in the Th1/Th2 balance by their direct effect on T cells. A shift in the Th1/Th2 balance may dysregulate the immune homeostasis and can lead to the breakdown of self-tolerance, precipitating autoimmunity [24,37,40]. To study the relationship between statin use and different aspects of the immune system, we assessed whether statins influenced the immune responses, by measuring CRP, neopterin levels, IgE antibodies and some prevalent autoantibodies (IgM rheumatoid factor (RF) and antinuclear antibodies (ANA)) in subjects from the general population who were either statin user or non-user. 

## Materials and Methods

### Study setting

Subjects from The Dutch Doetinchem Cohort Study were linked to the PHARMO Record Linkage System (PHARMO-RLS), using information on gender, and date of birth and postal code in order to obtain information on the use of statins to study markers representative of the immune status.

The Doetinchem Cohort Study is a population-based longitudinal study among inhabitants of the Dutch town Doetinchem. The main objective of this ongoing cohort study is to investigate the impact of (changes in) lifestyle and biological risk factors on the incidence of chronic diseases. A total of 12,405 men and women aged 20-59 years at baseline were examined in the years 1987-1991 (round 1) and a two-third random sample of these participants has been invited for (re-)examinations at 5-year intervals, during 1993-1997 (round 2), 1998-2002 (round 3) and 2003-2007 (round 4). Details on The Doetinchem Cohort Study have been previously described [41]. We used data from all four examination rounds of The Doetinchem Cohort Study. At every examination round demographic, lifestyle and health characteristics were collected using a self-administered questionnaire, including items regarding smoking and alcohol habits, educational level and physical activity. Additionally, participants underwent physical examination which included anthropometric and blood pressure measurements, and collection of blood samples. The Doetinchem Cohort Study was approved according to the guidelines of the Helsinki Declaration by the external Ethics Committee of the Dutch TNO Research Institute. Linkage between The Doetinchem Cohort Study and PHARMO-RLS has been conducted for subjects with an agreement in their informed consent [41].

PHARMO-RLS is a population-based database, comprising pharmacy-dispensing and hospital admission data of approximately 2.3 million community-dwelling inhabitants of 48 geographically defined areas in the Netherlands from 1985 onwards. With regard to prescription drugs, pharmacy records are virtually complete since the majority of Dutch inhabitants are registered with a single community pharmacy. For the present study, we used drug-dispensing and hospitalisation data. The pharmacy dispensing records include information on the type of drug dispensed, dispensing date, amount dispensed and prescribed dosage regimen. Hospitalisation data include detailed information on the primary and secondary discharge diagnoses, and dates of hospital admission and discharge. Drugs were coded according to the Anatomical Therapeutic Chemical (ATC) classification, and hospital diagnoses were coded according to the ICD-9-Cm codes [42,43]. Within these linked datasets, each subject was followed from the date of the first prescription of statins until collection of serum during examination.

### Study population

**Figure 1:**
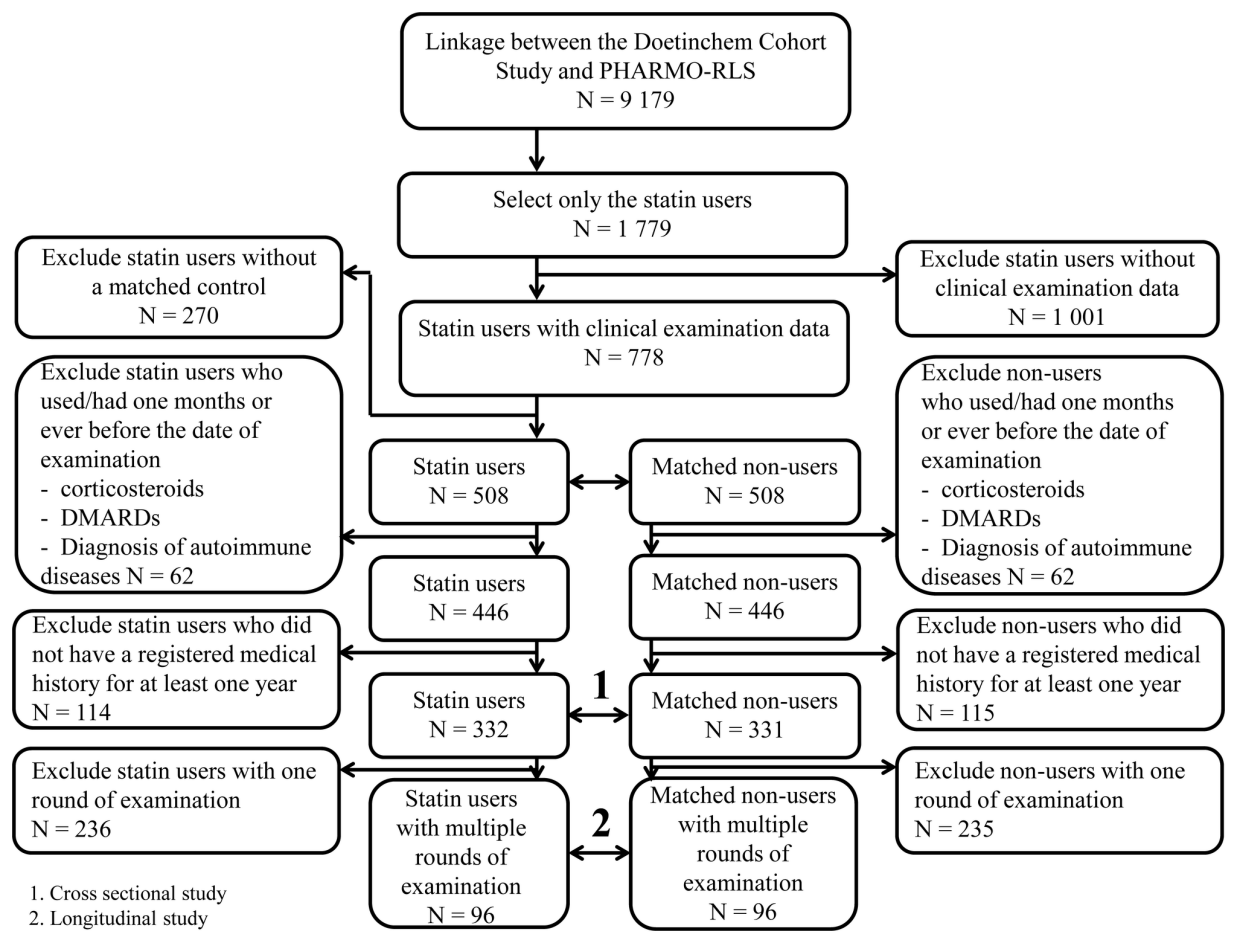
Study design.

Linkage between The Doetinchem Cohort Study and PHARMO-RLS has been conducted for 9,179 subjects. Of these subjects, 1,779 were statin users. Clinical examination data were available for 778 statin users. For each statin user, one non-user of statins was randomly selected and matched for age (+/-5 years), sex, date of examination, and history of cardiovascular diseases, diabetes mellitus type II and stroke. After matching on these factors, 1,016 subjects were included in the analysis. For the purpose of the study, subjects who used corticosteroids (n=22) one month before the date of examination, or who ever used disease modifying anti-rheumatic drugs (DMARD) (n=29) or had a diagnosis of an autoimmune disease before the date of examination were excluded, as were subjects who did not have a registered medical history for at least one year (n=73) at entrance of the linked data from The Doetinchem Cohort Study and PHARMO-RLS. Of these 892 subjects, 229 blood samples could not be retrieved. A total of 663 subjects (332 users and 331 non-users of statins) remained for the first analysis. For the second analysis, we selected from these 663 subjects, 192 subjects for whom blood samples were available from multiple rounds of examination. The study design is depicted in Figure 1. 

### Exposure definition

Exposure to statins was defined as the use of at least one prescription of any approved and commercially available statin (pravastatin, simvastatin, rosuvastatin, atorvastatin, fluvastatin) in the Netherlands before each date of examination. Cerivastatin was also included in this study, although cerivastatin was withdrawn from the market in the year 2001. Cumulative duration and daily dose of statins until examination was assessed, as well as the sum of prescriptions, defined daily doses (DDD) and adherence to statins [26]. Adherence to statin use was calculated by dividing the sum of the days' supply by the total number of days between the first prescription and the last prescription of statins in the year before the date of examination, multiplied by 100%. To determine adherence to statin use, we excluded subjects who received one prescription of statins, and who used no statin in the year before the date of examination. Subjects who received one prescription of statins before the date of examination were not excluded from other analyses. The potency of statins was determined by taking type and dose of statins into account, in order to control for the fact that different types and doses of statins differ with respect to the percentage reduction in low-density lipoprotein (LDL) cholesterol [44].

### Outcome definition

Non-fasting venous blood samples were drawn in each examination round. Blood samples were then centrifuged and separated sera were stored in 0.5 mL aliquots at -20°C until analyses. High sensitivity CRP was determined on a clinical autoanalyser (LX20-Pro, Beckman Coulter, Mijdrecht, The Netherlands), using standard kits. Data are expressed as mg/L. CRP levels lower than 2 mg/L are considered as normal [45]. Serum concentration of neopterin was measured by commercially available enzyme-linked immunosorbent assay (ELISA) kits (IBL, Hamburg, Germany, distributed via Mediphos, Renkum, The Netherlands) according to manufacturer’s instructions [46]. Data are expressed as ng/mL. Neopterin levels lower than 2.5 ng/mL were considered as normal (manufacturer’s recommendation). Levels of IgE antibodies were measured by using an Immunocap 250 (Phadia, Uppsala, Sweden), following manufacturer’s instructions. Data are expressed as IU/mL. Total IgE levels below 100 IU/mL were considered normal [47]. Sera were diluted 1:80 with phosphate buffered saline (PBS), and were screened for ANA by indirect immunofluorescence assay on HEp-2000 cells (ImmunoConcepts, Sacramento, CA, distributed via BMD, Antwerpen, Belgium) [48]. IgM-RF was determined with an ELISA (Sanquin Reagents, Amsterdam, the Netherlands), using a previously described protocol [49]. Serum levels above 12.5 IU/mL were considered positive based on measurements in a group of normal healthy blood donors in the Netherlands (n=665), which yielded IgM-RF values below 12.5 IU/mL in 96% of the cases according to manufacturer’s instructions [49]. The intra-assay and interassay variability were less than 10% for all test systems. To eliminate interassay variability, all samples from one patient were tested in a single assay. 

### Potential confounding variables

The following variables were considered as potential confounders: body mass index (BMI), educational level, smoking and drinking behaviour, physical activity level, parental history of myocardial infarction (MI), levels of total cholesterol and high-density lipoprotein (HDL) cholesterol, hypertension and asthma, and the use of non-steroidal anti-inflammatory drugs (NSAIDs), corticosteroids, proton pump inhibitors (PPIs), aspirin, antihypertensive and anti-diabetic drugs, antibiotics, antidepressants and hormone replacement therapy in the 6 months prior to the date of examination [50]. Parental history of MI was divided into two variables: 1) father with a history of MI and 2) mother with a history of MI, both with an age onset of <60 years. Hypertension was considered present when the subject had a systolic blood pressure of 140 mmHg or higher, a diastolic blood pressure of 90 mmHg or higher and/or used antihypertensive drugs. The matching factors age, sex, date of serum collection, and a history of cardiovascular diseases, diabetes mellitus type II and stroke were included in all models. In addition, all other co-variables that changed the regression coefficient for statin use by ≥10% were entered into the model [51].

### Statistical analysis

Baseline characteristics were reported as mean (SD) for continuous variables and proportion (percentage) for categorical variables. At baseline, comparisons between the non-users and users of statins were analysed using independent t and Mann-Whitney tests for continuous variables, and chi-square and Fisher exact tests for categorical variables. Skewed data were transformed logarithmically to approximate normal distribution. Distribution of levels of neopterin, IgE antibodies and CRP were positively skewed, and are presented as median with interquartile ranges (IQR). 

The associations between statin use and CRP and neopterin levels were studied in a linear regression model with adjustment for age, sex, date of examination, history of cardiovascular diseases, diabetes mellitus type II and stroke (model 1). In the second model lifestyle factors and cardiovascular disease risk factors were added, namely smoking status, antihypertensive drugs and total cholesterol levels. Similarly, the association of statin use with IgE levels was analysed with additional adjustment for asthma. Log-transformed markers CRP, neopterin and IgE antibodies were back transformed for presentation and the associations are expressed as slope (ß) with confidence intervals (CIs), representing the expected percentage change in the outcome [52]. Associations between statin use and the presence of elevated levels of CRP, neopterin and IgE, and ANA and IgM-RF were calculated using logistic regression models, analyses were adjusted for the same variables, as listed previously. The associations are expressed as odds ratio (OR) with CIs. We evaluated effects of cumulative duration and daily dose, DDDs, number of prescriptions, adherence and potency of statins on the immune status. To test *P* for trend of the associations across increasing quartiles of cumulative duration and daily dose, DDDs, number of prescriptions, adherence or potency of statins, the median values of these different exposure aspects of statin use were assigned to each quartile and used as a continuous variable in the linear regression model. 

Because of a potential modifying effect due to the presence of cardiovascular diseases, the analysis was stratified according to history of cardiovascular diseases. Changes in the immune system with aging and sex differences have been reported. Therefore, age- (<50, ≥50 years) and sex-stratified analyses for the evaluation of effect modification were carried out [53,54]. 

To study the relationship between statin use and the levels of CRP and IgE antibodies during follow-up, we used linear mixed-effects models for the analysis of repeated measurements with adjustment for the matching and baseline co-variables, as described above. The model deals with the correlation between repeated measurements in a subject, and allows subjects to have unequal gaps and numbers of observations. Only subjects with at least two serological measurements were included in the model. The random effects of the model include a random intercept and/or slope of time. In the model where we only included a random intercept, specification of a random slope did not change the results in a relevant way. Data are presented as ß’s with CIs and denotes the adjusted percentage change in the levels of CRP and IgE antibodies compared between and within statin users and non-users. *P*<0.05 was considered statistically significant using a 2-tailed test. Data were missing on several variables as listed in table 1. The missing values were imputed by the multiple imputation method by using the Markov Chain Monte Carlo Method [55]. All original outcome and co-variables presented in table 1 were included in the imputation model. Twenty imputation sets were created, analysed and pooled by the MIANALYZE procedure. Baseline measurements of complete and imputed cases were compared based on the means and frequencies. Results from the complete case and multiple imputation analyses were compared and multiple imputation analyses are presented. All analyses were performed using SAS version 9.2 (SAS Institute, Cary, NC).

**Table 1:** Baseline characteristics of the study population.

	Statin users	Statin users	Non-users	Non-users	p-value
	N	(n=332)	N	(n=331)	
Men (%)	332	196 (59)	331	194 (59)	-
Mean age in years (SD)	332	59.5 ± 8.0	331	59.3 ± 8.0	-
Mean BMI in kg/m^2^(SD)^a^	316	26.8 ± 3.4	292	26.1 ± 3.6	0.02
Low education level (%)	253	136 (54)	236	131 (56)	0.72
History myocardial infarction father (%)	316	41 (13)	292	26 (9)	0.11
Current smoking (%)	252	74 (29)	236	57 (24)	0.10
Regular alcohol consumption (%)	299	193 (65)	271	186 (69)	0.61
Physical active (%)	236	188 (80)	216	156 (72)	0.06
Mean total cholesterol in mmol/L (SD)	316	6.82 ± 1.18	291	5.68 ± 1.01	<0.0001
Mean HDL cholesterol in mmol/L (SD)^b^	316	1.19 ± 0.32	291	1.30 ± 0.35	<0.0001
Disease history before date of examination (%)					
Diabetes	266	67 (25)	249	50 (20)	-
Hypertension	332	147 (47)	331	99 (34)	0.002
Cardiovascular disease	332	87 (26)	331	25 (8)	<0.0001
Stroke	315	11 (3)	290	9 (3)	-
Asthma	261	5 (2)	245	10 (4)	0.15
Cancer	332	16 (5)	331	15 (5)	0.86
Drug use 6 months before date of examination (%)					
Corticosteroids	332	3 (1)	331	3 (1)	1.00
NSAIDs^c^	332	44 (13)	331	53 (16)	0.31
Fibrates	332	1 (0)	331	0 (0)	1.00
Antihypertensive drugs	332	135 (41)	331	53 (16)	<0.0001
Anti-diabetic drugs	332	29 (9)	331	23 (7)	0.39
Aspirin	332	78 (24)	331	15 (5)	<0.0001
HRT^d^	332	12 (4)	331	10 (3)	0.67
Antibiotics	332	32 (10)	331	26 (8)	0.42
Antidepressants	332	17 (5)	331	18 (5)	0.85
PPIs^e^	332	26 (8)	331	14 (4)	0.05

^a^ BMI = Body mass index

^b^ HDL = High density lipoprotein

^c^ NSAIDs = Nonsteroidal anti-inflammatory drugs

^d^ HRT = Hormone replacement therapy

^e^ PPIs = Proton pump inhibitors

## Results

### Baseline characteristics

Baseline characteristics of the study population are presented in table 1. The study comprised 332 statin users of whom 196 (59%) were men with a mean age of 59.5 (SD 8.0) years and 331 non-users of whom 194 (59%) were men with a mean age of 59.3 (SD 8.0) years. In statin users, total cholesterol levels and BMI were higher and the level of HDL cholesterol was lower than in non-users. Hypertension and cardiovascular disease were more frequently reported among statin users than non-users. As a result of these differences, antihypertensive drugs, aspirin and PPIs were more used among statin users. 

### Inflammation

Measurements of immune parameters are presented in Table 2. The median (IQR) level of CRP was significantly lower for statin users, 1.28 (0.59-2.79) mg/L, than for non-users 1.62 (0.79-3.35) mg/L. After controlling for the matching variables, antihypertensive drugs, total cholesterol levels and smoking status, a lower CRP level was observed in statin users compared with non-users (difference of -28% for statin use vs. non-use, *P*<0.01). When we used a cut-off value of less than 2 mg/L to discriminate between normal and increased levels of CRP, statin use was associated with the presence of low levels of CRP (OR: 0.67; 95% CI: 0.45-0.99). 

**Table 2:** Regression coefficients^a^ and odds ratios for the association between statin use and serum immune markers in the general population.

	Non-users	Statin users			
	N=331	N=322			
	Median (IQR)	Median (IQR)	Crude β(95% CI)^c^	Adjusted β(95% CI)^d^	p-value^f^
CRP (mg/L)^b^	1.62 (0.79-3.35)	1.28 (0.59-2.79)	0.79 (0.64;0.97)	0.72 (0.56;0.91)	0.007
Neopterin (ng/mL)^b^	1.83 (1.45-2.21)	1.77 (1.45-2.26)	0.98 (0.93;1.03)	0.96 (0.90;1.03)	0.26
IgE (IU/mL)^b^,^e^	25.30 (10.50-62.40)	28.60 (11.40-79.65)	1.16 (0.95;1.42)	1.20 (0.98;1.51)	0.09
	N (%)	N (%)	Crude OR (95% CI)	Adjusted OR (95% CI)	p-value
CRP (> 2 mg/L)	119 (46.1)	213 (52.6)	0.75 (0.54;1.03)	0.67 (0.45;0.99)	0.05
Neopterin (>2.5 ng/mL)	30 (9.1)	33 (9.9)	0.95 (0.55;1.63)	0.92 (0.49;1.75)	0.81
IgE (>100 IU/mL)^e^	47 (14.2)	66 (19.9)	1.54 (1.00;2.36)	1.68 (1.01;2.79)	0.05
ANA	40 (12.1)	38 (11.5)	0.83 (0.51;1.35)	0.76 (0.42;1.37)	0.36
IgMRF (>12.5 IU/mL)	31 (9.4)	29 (8.7)	0.94 (0.55;1.63)	0.96 (0.50;1.85)	0.91

IQR, interquartile range; CRP, C-reactive protein; IgE, Immunoglobulin E; ANA, antinuclear antibodies; IgM-RF, IgM-Rheumatoid factor

^a^ Regression coefficients denote the adjusted percentage difference in serum CRP levels (mg/L) of statin users compared to non-users (reference group).

^b^ Differences of the geometric means were reported for CRP, neopterin and IgE levels as these variables were non-normally distributed and the natural logarithm was used in the analyses.

^c^ Adjusted for the matching co-variables age, sex, date of examination, cardiovascular diseases, diabetes mellitus type II and stroke (model 1).

^d^ Adjusted for the matching co-variables from model 1 and antihypertensive drugs, smoking and total cholesterol levels (model 2).

^e^ Adjusted for the co-variables from model 1 and 2, and asthma

^f^ P < 0.05 after adjustment for confounding factors.

### Immunomodulation

We found no difference in levels of neopterin or the prevalence of low levels of neopterin (<2.5 ng/mL) between statin users and non-users. The median IgE level in statin users was 28.6 IU/mL compared to 25.3 IU/mL in non-users. The prevalence of elevated levels of IgE antibodies (>100 IU/mL) was higher in statin users compared to non-users. A trend towards higher levels of IgE antibodies was observed in statin users compared with non-users, although this difference did not meet the criteria for significance. 

### Self-tolerance

No associations were observed between statin use and the presence of the autoantibodies, ANA and IgM-RF.

### CRP and IgE levels and different aspects of exposure to statins

As we observed associations between statin use and levels of CRP and IgE antibodies, we studied these associations in detail in statin users by the different aspects of exposure to statins. We present in table 3 the regression coefficients for the different aspects of exposure to statins in relation to CRP and IgE levels, adjusted for confounders. Regarding the number of prescriptions, cumulative duration and daily dose, DDDs, potency and adherence to statins, associations with CRP levels were inverse, although none of them were statistically significant. In addition, an association between increasing number of days of statin use and decreased levels of CRP was observed (*P*_for trend_=0.02). For IgE levels, the fully adjusted associations with different aspects of exposure to statins were positive, but no association was statistically significant. 

**Table 3:** Association between different aspects of statin use and levels of CRP and IgE, presented by the number of prescriptions, cumulative duration, cumulative daily dose and defined daily dose (DDD), potency and adherence.

		Levels of CRP (mg/L)^a^,^b^					Levels of IgE (IE/mL)		
	Statin users	Crude β(95% CI)^c^	Adjusted β(95% CI)^d^	*P*_trend_^f^		Statin users	Crude β(95% CI)	Adjusted β(95% CI)^d,e^	*P*_trend_^f^
	N=332					N=332			
No. of prescriptions									
0	331	Reference	reference			331	reference	reference	
1-15	116	0.85 (0.63;1.14)	0.78 (0.57;1.07)			116	1.38 (0.98;1.84)	1.38 (0.99;1.88)	
16-30	112	0.62 (0.46;0.84)	0.56 (0.40;0.77)			112	1.16 (0.87;1.55)	1.18 (0.86;1.63)	
≥31	104	0.91 (0.68;1.22)	0.81 (0.58;1.13)	0.20		104	0.98 (0.74;1.31)	1.00 (0.72;1.39)	0.70
Cumulative duration,days									
0	331	Reference	reference			331	reference	reference	
1-550	125	0.87 (0.66;1.15)	0.78 (0.58;1.05)			125	1.22 (0.93;1.59)	1.23 (0.92;1.66)	
551-1100	78	0.75 (0.85;1.05)	0.68 (0.48;0.97)			78	1.36 (0.98;1.88)	1.38 (0.97;1.96)	
≥1101	129	0.72 (0.55;0.96)	0.67 (0.49;0.92)	0.02		129	1.01 (0.77;1.32)	1.03 (0.76;1.41)	0.98
Cumulative daily dose^g^									
0	331	reference	reference			331	reference	reference	
1-1800	122	0.82 (0.62;1.08)	0.77 (0.57;1.03)			122	1.29 (0.98;1.69)	1.30 (0.97;1.74)	
1801-3600	83	0.66 (0.47;0.92)	0.57 (0.40;0.81)			83	1.26 (0.91;1.74)	1.28 (0.90;1.83)	
≥3601	127	0.85 (0.64;1.12)	0.77 (0.56;1.06)	0.12		127	1.00 (0.76;1.31)	1.01 (0.74;1.38)	0.83
DDD									
0	331	reference	reference			331	reference	reference	
≤1	123	0.73 (0.55;0.96)	0.69 (0.49;0.97)			123	1.07 (0.81;1.44)	1.11 (0.82;1.49)	
1.1-1.5	94	0.75 (0.55;1.02)	0.67 (0.50;0.91)			94	1.18 (0.87;1.60)	1.22 (0.87;1.70)	
≥1.6	115	0.90 (0.67;1.21)	0.79 (0.57;1.09)	0.06		115	1.26 (0.81;1.40)	1.30 (0.94;1.78)	0.10
Potency^h^									
0	331	reference	reference			331	reference	reference	
1-30	92	0.79 (0.58;1.08)	0.71 (0.51;1.01)			92	1.33 (0.98;1.80)	1.36 (0.98;1.89)	
31-35	99	0.76 (0.56;1.03)	0.70 (0.50;0.98)			99	1.18 (0.88;1.58)	1.21 (0.87;1.68)	
≥36	139	0.81 (0.61;1.07)	0.72 (0.53;0.98)	0.10		139	1.07 (0.82;1.40)	1.10 (0.82;1.48)	0.20
Adherence^i^									
0	298	reference	reference			298	reference	reference	
1-50	51	0.61 (0.41;0.91)	0.53 (0.35;0.80)			51	1.52 (1.03;2.23)	1.53 (0.99;2.28)	
51-80	53	1.09 (0.74;1.61)	0.89 (0.59;1.35)			53	0.98 (0.67;1.43)	0.97 (0.64;1.46)	
81-100	194	0.71 (0.55;0.99)	0.66 (0.50;0.88)	0.16		194	1.09 (0.86;1.39)	1.11 (0.84;1.46)	0.71

CRP, C-reactive protein; IgE, Immunoglobulin E

^a^ Regression coefficients denote the adjusted percentage difference in difference in serum CRP levels (mg/L) of statin users compared to non-users (reference group).

^b^ Differences of the geometric means were reported for CRP and IgE levels as these variables were non-normally distributed and the natural logarithm was used in the analyses.

^c^ Adjusted for the matching co-variables age, sex, date of examination, cardiovascular diseases, diabetes mellitus type II and stroke (model 1).

^d^ Adjusted for the matching co-variables from model 1 and antihypertensive drugs, smoking and total cholesterol levels (model 2).

^e^ Adjusted for the co-variables from model 1 and 2, and asthma

^f^ *P*(<0.05) for trend refers to a linear trend in regression coefficients across increasing categories of the number of prescriptions, cumulative duration, cumulative daily dose and DDD, adherence or potency of statins by treating the median of each category as a continuous variable in the model.

^g^ Cumulative statin dose was defined as the total defined daily dose of drugs prescribed prior to the date of examination.

^h^ The potency of statins was determined by taking type and dose of statins into account, in order to control for the fact that different types and doses of statins differ with respect to the percentage reduction in LDL-cholesterol.

^i^ Adherence in the year prior to the date of examination was the sum of the days’ supply, divided by the total number of days between the first and last prescription of statins multiplied by 100%. Subjects who received only one prescription of statins and who used statins more than one year before the date of examination were excluded from the study, and therefore the total number of subjects in the analysis is 629 (298 statin users and 331 controls).

### CRP and IgE levels and different groups of age, sex and history of cardiovascular diseases

The associations between statin use and levels of CRP did not differ between strata of sex, age and history of cardiovascular diseases (data not shown). However, levels of IgE antibodies were higher in statin users versus non-users who were older than 60 years and had a history of cardiovascular disease (data not shown). 

### Development of CRP and IgE levels over time

Cross-sectional analyses did not show differences in the levels of neopterin, and the presence of ANA and IgM-RF between statin users and non-users. Therefore, we only studied the change in CRP and IgE levels over time between statin users and non-users (table 4). After controlling for the matching variables, antihypertensive drugs, total cholesterol levels and smoking status, the change over time in mean CRP level was lower in statin users compared to non-users, by on average 29% (*P*=0.04). No difference in the change over time in mean levels of IgE antibodies between statin users and non-users was observed. We found no effect modification between time and statin treatment between users and non-users of statins, indicating that the difference in CRP levels remained constant over time.

**Table 4:** Regression coefficients^a^ for the change in CRP and IgE levels over time between statin users and non-users.

	Crude β(95% CI)^b,c^	Adjusted β(95% CI)	p-value^g^
CRP (mg/l)^d^,^f^	0.81 (0.98; 1.07)	0.71 (0.51; 0.98)	0.04
IgE (IE/ml)^e^	0.87 (0.84; 1.41)	0.97 (0.84; 1.13)	0.70

CRP, C-reactive protein; IgE, Immunoglobulin E

^a^ Regression coefficients denote the adjusted percentage change in the levels of CRP and IgE antibodies compared between and within statin users and non-users (reference group).

^b^ Differences of the geometric means were reported for CRP and IgE levels as these variables were non-normally distributed and the natural logarithm was used in the analyses.

^c^ Adjusted for the matching co-variables age, sex, date of examination, cardiovascular diseases, diabetes mellitus type II and stroke (model 1).

^d^ Adjusted for the matching co-variables from model 1 and antihypertensive drugs, smoking and total cholesterol levels (model 2).

^e^ Adjusted for the co-variables from model 1 and 2, and asthma

^f^ The random-effects portion of the model consists only of a random intercept.

^g^ P < 0.05 after adjustment for confounding factors.

## Discussion

In the present study, we observed that statins suppress the innate immune response, by decreasing the levels of CRP, both cross-sectionally and over time. A more detailed analysis on different aspects of exposure to statins, showed an inverse association between duration of statin use and levels of CRP. Furthermore, we observed a trend towards higher levels of IgE antibodies in statin users, which infers that it did not meet the pre-specified criteria for significance. Neopterin levels were not affected. Finally, ANA and IgM-RF were not different between statin users and non-users suggesting that the use of statins was not associated with a loss of self-tolerance. 

Importantly, our study consistently showed that the use of statins is associated with decreased levels of CRP in the general population, as previously described in population-based studies and clinical trials [33,56-60]. Essentially, by confirming this well-known association between statin use and decreased levels of CRP, it demonstrates that we have used appropriate methods for patient selection. This strengthens the conclusions of our current study.

Apart from the anti-inflammatory effects, it has been suggested that statins may have a direct immunomodulating effect on T cells and may promote a shift from Th1 to Th2 immune responses, possibly leading to a dysregulation in the immune homeostasis [24,30,37]. Cherfan et al. reported that statins did not affect Th1 cells but suppressed Th2 responses [58]. In contrast, other studies suggested that statins promote a Th2 bias [61,62]. In our study, we observed a trend towards increased levels of IgE antibodies, representative of Th2 responses, but this was not accompanied by a decrease in neopterin levels, a marker for Th1 cell activity. This is in agreement with two other studies showing that treatment with statins had no effect on the levels of neopterin [63,64]. However, two studies including patients with cardiovascular diseases showed that neopterin levels declined during statin treatment [36,65], indicating a suppressive effect on Th1 cells. We found no association between statin use and the presence of the most prevalent autoantibodies, ANA and IgM-RF [66,67], suggesting maintenance of self-tolerance. So far, an indication for an association between statin use and the presence of ANA was shown in case reports of lupus-like syndrome, poly- and dermatomyositis [24]. To date, no study was performed on statin use and the presence of RF in the general population. The study of Chodick, et al. actually showed a decreased risk of RA in persistent statin users [7]. In this study, persistent statin users were compared to non-persistent statin users and were followed until the study outcome (RA). In our current study, we compared statin-users with non-statin users. The outcome variable autoimmune disease, i.e. RA, was not part of our study. We measured incidence of IgM-RF, in combination with ANA, merely as markers for loss of self-tolerance. However, when the risk of RA was compared between statin users and non-users, three population-based studies demonstrated no association between statin use and the risk of developing RA [9,68,69].

Recently, Mammen et al. identified the presence of anti-HMGCR antibodies in statin-associated autoimmune myopathy [70]. The same authors screened a large population of statin exposed patients without myopathy and found no anti-HMGCR antibodies in these subjects [71]. As our observations showed that statins were not related to the development of autoantibodies in the general population, we hypothesise that statins do not themselves cause autoimmunity but that they may promote a pre-existing autoimmune-prone condition to progress towards clinically manifest diseases. Several studies demonstrated that statins may exacerbate or trigger cellular apoptosis [24,72,73], which may lead to increased levels of apoptotic cells and blebs, resulting in increased amounts of autoantigens in the circulation [74,75]. As patients treated with statins may be less efficient in clearing apoptotic cells, they may be more susceptible to autoimmune diseases. Furthermore, statins may be able to amplify autoimmunity in genetically susceptible individuals, thereby increasing the risk of developing autoimmune diseases [76]. It is known that statins not only interfere with the mevalonate or cholesterol synthesis pathway but also inhibit the prenylation of Rho GTPases, including Rac-1 [77,78]. Rac-1 is directly implicated in the activation of caspase-1 [79], which is crucial for processing and secretion of the proinflammatory cytokines IL-1β and IL-18, promoting autoimmunity [80,81]. However, patients with mutations in the mevalonate kinase gene (e.g. hyper-IgD syndrome) may respond differently to the treatment with statins [82], although the prevalence rate of this immune disorder is very low. To prove our hypothesis, however, a much larger cohort study (probably more than 40,000 subjects [83]) should be performed. 

One of the strengths of this study is the linkage between a large population-based cohort study and a registry of pharmacy and hospital discharge records, enabling us to assess data on statin use and sera from subjects in Doetinchem. Another strong feature of our study is the prospective and longitudinal design with a relatively long follow-up (>15 y). Moreover, as sera were collected at several time points, our subjects were compared not only cross-sectionally but also longitudinally over time. Using a computerised database, PHARMO-RLS, we were allowed to use routinely recorded dispensing data from pharmacies. Consequently, recall and non-response bias were minimised. Furthermore, detailed information on confounders, including smoking status was available.

Some limitations of our study should be acknowledged. First, we have selected a restricted number of immune markers in our study as statins may have influenced other T-cell-mediated responses. It has been suggested that statins block the differentiation of the pro-inflammatory Th17 cells and have a direct effect on regulatory T cells (Treg) in atherosclerotic plaques in mice and peripheral circulation of coronary patients [84-86]. Regrettably, to date, no valid markers for detecting Th17 and Treg cells in human sera are available. Furthermore, the long-term storage of our samples limited us in the selection of immune markers as our samples were stored from 2 up to 20 years (depending on the round of examination). Long-term storage of the sera samples may have affected the levels of various immune markers, such as antibodies, chemokines, cytokines and other soluble markers of immune activation. However, several studies have shown that prolonged storage did not affect CRP [87], neopterin [88] and IgE antibodies [89]. Since statin users and non-users were matched on date of examination in order to maintain an equally distributed duration of sample storage, we do not expect that long-term storage influenced our associations between statin use and the levels of CRP, neopterin and IgE antibodies. Second, despite our best efforts, matching on cardiovascular diseases was only partially achieved as the majority of the subjects with cardiovascular diseases were treated with statins. However, when we stratified for cardiovascular diseases, no differences in the association between statin use and immune markers were observed. Third, statin users or non-users may have visited a pharmacy, not participating in our computerised database to collect their prescription, possibly resulting in an underestimation of the actual statin use. However, a recent study showed that the majority of Dutch patients are loyal to their pharmacy, i.e., approximately only 11% of patients visited two or more pharmacies in 2001 [90]. When patients visited another pharmacy, it would most likely be a pharmacy in the same region. Since we had data from all pharmacies in the Doetinchem region, we could assess the complete medication history from a single statin user or non-user across these pharmacies. Therefore, we do not expect that this would have affected our findings.

Fourth, the linkage of The Doetinchem Cohort Study and the PHARMO-RLS database yielded 663 subjects who had at least one serum sample in one of the examination rounds. This restricted number of subjects may have raised concerns about the generalisability of our findings. It is conceivable that this may have affected the estimates of the baseline characteristics, but we believe to a lesser extent on the magnitude of the associations between statin use and the serological markers of (auto) immunity. Finally, it should be acknowledged that this is an observational study which may be subjected to residual confounding due to potential unmeasured differences in cardiovascular risk profile and subjects’ characteristics between statin users and non-users. However, we have matched the statin users and non-users on several factors; we believe we have reduced residual confounding as much as possible.

## Conclusions

In the present study, we have demonstrated that statins exert anti-inflammatory effects as shown by a decrease in levels of CRP. However, no effects on the levels of neopterin and the presence of the autoantibodies, ANA and RF, were shown. These findings suggest that this population-based study does not provide evidence that statins exert immunomodulatory effects on a Th1 response and/or loss of self-tolerance. An immunomodulatory effect on Th2 response, however, cannot be excluded as we observed a slight increase in IgE levels. Further research is warranted to investigate the immunomodulatory effects of statins in peripheral blood of healthy individuals from a large population-based cohort study.
